# Topical interventions for the management of pain in chronic wounds: A protocol for a systematic review

**DOI:** 10.12688/hrbopenres.13560.1

**Published:** 2022-08-19

**Authors:** John D. Ivory, David P. Finn, Akke Vellinga, Karen Butler, Duygu Sezgin, Aonghus O'Loughlin, Peter Carr, Catherine Healy, Georgina Gethin

**Affiliations:** 1 School of Nursing & Midwifery, University of Galway, Galway, H91TK33, Ireland; 2 Alliance for Research and Innovation in Wounds (ARIW), University of Galway, Galway, H91TK33, Ireland; 3 Irish Research Council (IRC), Dublin, D04C2Y6, Ireland; 4 Pharmacology & Therapeutics, School of Medicine, University of Galway, Galway, H91TK33, Ireland; 5 Galway Neuroscience Centre, University of Galway, Galway, H91TK33, Ireland; 6 Centre for Pain Research, University of Galway, Galway, H91TK33, Ireland; 7 School of Public Health, Physiotherapy and Sports Science, University College Dublin, Dublin, D04V1W8, Ireland; 8 School of Medicine, University of Galway, Galway, H91TK33, Ireland; 9 University Hospital Galway, Galway, H91YR7, Ireland; 10 School of Nursing & Midwifery, Monash University, Melbourne, Victoria, 3800, Australia

**Keywords:** pain, chronic wound, wound bed, topical agent, dressings, systematic review

## Abstract

**Background:** Venous, arterial, diabetic and pressure ulcers, collectively known as chronic wounds, negatively impact individuals across psychological, social and financial domains. Chronic wounds can be painful and the nature, frequency and impact of pain can differ depending on wound aetiology, wound state and on numerous patient factors. While systemic pharmaceutical agents have some effect in managing pain, there is a need to examine topical agents applied to the wound bed for pain relief. The objective of this study is to examine and synthesise existing literature on the effectiveness of topical agents in managing pain in venous, diabetic, pressure, arterial and mixed venous/arterial ulcers.

**Methods:** We will use Cochrane Systematic Review methodology to identify and synthesise eligible randomised controlled trials (RCTs) evaluating the effectiveness of topical agents in reducing pain in chronic wounds. Embase, Medline, PubMed, CENTRAL, CINAHL, Scopus and Web of Science will be searched from inception to end of June 2022 without language limits. We will independently extract data on the pharmaceutical agent, participant demographics, aetiology, condition of the wound, and type, nature and frequency of pain using a pre-designed data extraction form. Subgroup and sensitivity analysis will be performed to address heterogeneity across studies if appropriate. Further stratification and analyses will be based on included study variables and outcomes.

**Discussion: **Wound pain is primarily managed *via* systemic pharmaceutical agents. However, patients express reluctance regarding systemic analgesic drugs, fearing addiction. Additionally, persons with chronic wounds have co-morbidities including hypertension, diabetes, or cardiovascular disease and are already taking multiple medications. Topical analgesia can potentially mitigate some of the perceived disadvantages of systemic agents but the available range of these agents and their effectiveness in managing pain in chronic wounds is not so well understood. This review will focus on such agents across a range of the most common chronic wounds.

## Introduction

Under normal circumstances, trauma to skin repairs along a trajectory of orderly haemostatic, inflammatory, proliferative and remodelling/scar maturation phases ^1, 2^. However, when certain morbidities present, this ordered process can become disrupted, the wound stalls; usually in the inflammatory or proliferative phases and it will fail to heal in a timely fashion ^3, 4^. Hard-to-heal or chronic wounds occur secondary to, among others, conditions such as venous insufficiency, diabetes, arterial complications and prolonged pressure, friction or shear on skin surfaces, and they currently affect up to 2.21 per 1,000 population ^5^. They bring heavy burdens to individuals across multiple aspects of their lives including relationships, finances, work and contribution to society, and to society itself with chronic wounds costing the United Kingdom’s National Health Service (NHS) GBP 8.3 billion in 2017/18 ^6– 8^.

Pain is a significant element in the burden posed to the individual by chronic wounds with more than 80% of venous leg ulcer (VLU) patients in two studies reporting acute or chronic pain in the wound (half of these rated pain as moderate to the worst possible), and 59% of pressure ulcer patients in a third reporting pain from wounds in a hospital setting ^9^.

Heretofore, management of wound pain is primarily through systemic pharmaceutical agents. Discussions with our patient panel of the *Alliance for Research and Innovation in Wounds (ARIW)* reflects a reluctance to take more systemic analgesic drugs and a fear of addiction. Additionally, it is well reported that people with chronic wounds, many of whom are older adults, have at least one co-morbidity including hypertension, diabetes, or cardiovascular disease and are invariably already taking multiple medications ^10^. A recent systematic review reported a pooled prevalence of wound-related background pain in persons with chronic VLUs as 80% (95% CI 65–92%) with a mean pain intensity score of four (0–10 numeric rating scale) (95% CI 3.4–4.5) ^11^. The use of topical analgesia can mitigate some of the perceived disadvantages of systemic agents. At present, there is a lack of understanding of the range of topical agents that are available and their level of effectiveness in managing pain in chronic wounds. A previous systematic review has dealt solely with VLUs while this review will focus across a range of the most common chronic wounds ^12^.

The aim of this review is to address this gap in the literature, by examining and synthesising existing literature on the effects of topical agents in the management of pain in the most common types of chronic wounds and is therefore limited to venous, diabetic, pressure, arterial and mixed venous arterial ulcers.

### Research question

What are the effects of topical agents in the management of wound pain in patients with chronic wounds?

## Methods

This protocol is reported in line with the PRISMA-P guidelines ^13^.

### Eligibility criteria

***Studies.*** We will allow randomised controlled trials (RCTs) with any type of true random allocation method such as individual, stepped wedge or cluster entry to this review. We will deem quasi-randomised studies with allocation based on non-random methods such as birth date or alternation as ineligible for entry to this review.

***Participants.*** Our patient population will consist of adults (18 years old and over) with VLUs, diabetic foot ulcers (DFU), arterial ulcers, mixed arterial venous ulcers (MAVLU) or PUs. Appropriate care settings will include residential care facilities, hospitals, general practitioner offices, outpatient departments or the home setting. Wound diagnosis will be as per reporting author.

We will not include studies reporting on persons with acute wounds, burns, surgical wounds, malignant wounds, atypical wounds or pain experienced as a result of dressing changes. We recognise that these are also painful wounds but the underlying pathophysiology is different and therefore different approaches are often required for treatment.

We will include studies reporting more than one wound aetiology if any of those aetiologies meet our inclusion criteria and have results presented accordingly.

***Interventions.*** We will investigate topical agents indicated for treatment of wound pain by direct application to the wound bed. These agents may fall into the following groups:

- Pharmacological agents. These could include topical non-steroidal anti-inflammatory drugs (NSAIDs), opioids, local anaesthetics, capsaicin and/or cannabinoids.
- Non-pharmacological agents. These could include wound dressings and/or complementary/alternative therapies.

Where an intervention does not touch or interact with the wound bed it will be excluded.

***Outcome assessments***

Public and patient involvement

We consulted with individuals from the ARIW patient panel. We ultimately selected outcomes that best reflected the panel’s needs yet could be identified as meaningful in the literature with respect to assessing benefits and harms ^7, 14– 16^.

Main outcomes

- Any assessments of pain intensity measured on a continuous scale *e.g.*, numerical rating scales (NRS) or visual analogue scales (VAS).
- The proportion of participants with any reduction or improvement in pain intensity.

Additional outcomes

- The proportion of participants with ≥ 30% pre-to-post treatment reduction in pain intensity (equivalent to a moderate improvement defined by IMMPACT) ^15^.
- Reported changes in disability or physical functionality.
- Reported changes in emotional functionality or impact on mental health *e.g.*, anxiety, depression, mood, *etc*.
- Reported changes to quality-of-life score, measured using any quality-of-life assessment tool.
- Adverse events. For this review adverse events will include reported measures of harm, withdrawal because of adverse events or serious adverse events, patient reported adverse events, and specific adverse events - especially central nervous system (CNS) and cardiovascular. We will describe how adverse events were addressed, how they were reported, and over what time period the harm was experienced as per the PRISMA harms checklist ^17^.
- Rescue analgesia requirements *e.g.*, time to rescue.
- Patient-reported changes to sleep quality and duration.
- Analgesic effects onset and duration.
- Reported changes in cognitive functioning.

Included studies will compare their intervention to either a placebo or another intervention. We will collect information on all relevant outcomes reported in any given outcome category. In the event that an outcome is assessed by two or more outcome measures in the same study, two review authors will select the primary outcome measure as identified by the publication authors. Otherwise, they will select the measure specified in the sample size calculation and rank effect estimates *i.e.*, list them in descending order.

### Search strategy

We will conduct the literature search as follows. We will search Ovid MEDLINE (RRID:SCR_002185), PubMed (RRID:SCR_004846), EMBASE (RRID:SCR_001650), EBSCOhost CINAHL, The Cochrane Central Register of Controlled Trials (CENTRAL) (RRID:SCR_006576), Scopus and Web of Science (RRID:SCR_017657) from inception to the end of June 2022 without any language limits. We will search the ClinicalTrials.gov (RRID:SCR_002309) and EudracT trial registries. We will scan reference lists of included studies and identified systematic reviews. We will also search conference proceedings for unpublished work and contact authors where necessary.

The search strategy was developed *via* an iterative process with the PRESS Guideline Evidence-Based Checklist in mind ^18^. We examined previous, relevant literature and ran a series of sample searches across our chosen databases. Terms were organised to capture three distinct concepts: chronic wound aetiologies, pain secondary to chronic wounds and interventions to treat this pain. Terms relevant to each of these three constructs were finally combined with Boolean AND/OR operators to create the final strategy (see *Extended data*) ^13^. Additional filters limited the search to RCTs conducted in human populations.

### Data collection and analysis

***Data extraction (selection and coding).*** Review team members working in pairs (but independently of each other) will screen randomly allocated portions of titles and abstracts against clearly identified and pre-tested eligibility criteria using the online systematic review software package Rayyan QCRI (RRID:SCR_017584). Disagreements between screening partners will be resolved by discussion and input from a third party if necessary, and decisions will be made by consensus. We will retrieve the full text of any papers or reports identified as potentially relevant. Two review authors will independently screen full-text studies for inclusion or exclusion with discrepancies resolved by discussion with a third review author to reach consensus. All studies excluded from the review at this stage will be listed as excluded, with the reasons recorded.

To minimise differences between reviewers in the screening process, team members will perform pilot calibration exercises on a random sample of 100 references. They will apply the inclusion and exclusion criteria to a common set of titles and abstracts. The level of agreement (whether the articles were included or excluded) will then be calculated; the aim is to reach at least 90% agreement on the rating of a sample of references. This process of calibration will be repeated until the goal of at least 90% agreement is reached ^19^. We will document the screening process in a PRISMA flow chart and a ‘Characteristics of excluded studies’ table. We will document relevant information about ongoing studies including citation details, and we will also document details of multiple reports published on the same study to ensure that original studies and not reports are the units of interest for this review.

Review authors working in pairs (but independently of each other) will extract data from eligible studies. Disagreements will be resolved by discussion between pair members until consensus is reached or through consultation with a third review author if necessary. Review authors will not extract data from their own studies. We will develop and pilot a review-specific data extraction worksheet.

The worksheet will capture the following data:

- General study/publication information *e.g.*, journal, study title, corresponding author, year of publication, country of conduct.
- Study methodology information *e.g.*, study objective, methodological design.
- Study population information *e.g.*, participant characteristics including age, gender, co-morbidities, concurrent medications *etc*.
- Wound information *e.g.*, wound aetiology and condition, wound size, location, depth & duration.
- Outcome information *e.g.*, primary and secondary outcomes if reported and means of their assessment.
- Intervention/comparator information *e.g.*, intervention name, mode of delivery, dose and frequency of application.

A single review author will enter extracted data into Review Manager 2020 (Review Manager (RevMan) [Computer program], Version 5.4.1, The Cochrane Collaboration, 2020) (RRID:SCR_003581). A second author will independently check entered data for accuracy against the data extraction work sheets. Authors will resolve disagreements by discussion with a third author available to intervene if necessary.

***Risk of bias (quality) assessment.*** We will use the Cochrane Risk of Bias Tool (RoB2) to assess methods of included studies for risk of bias ^20^. The tool explores the following methodological domains: random sequence generation, allocation concealment, participant and personnel blinding, and outcome assessment blinding, outcome data completeness, selective reporting of outcomes, and other sources of bias. We will consider blinding by outcome where appropriate. We will consider outcome data completeness in terms of length of follow-up and will consider all outcomes in terms of time of assessment. We will determine each item in a study as having a low, unclear or high risk of bias, as per criteria reported in the Cochrane handbook for Systematic Reviews of Interventions ^21^. We will generate a risk of bias table and include it in the review. We will provide justification and quoted text for our judgement on each item in a study in the risk of bias table.

In addition to the risk of bias domains mentioned above, we will assess risk of bias from selective recruitment of participants for any included cluster RCTs. Two review authors will assess risk of bias in included studies independently and disagreements will be resolved by discussion between the authors. If necessary, a third author will intervene to facilitate consensus. We will contact study authors for clarification of study methods or for additional information when required.

We will assess quality of the evidence using the GRADE system ^22^. GRADE assesses evidence on four levels of quality: very low, low, moderate and high, based on factors such as study limitations, unexplained heterogeneity or inconsistency, imprecision, indirectness and publication bias for each outcome.

***Strategy for data synthesis.*** We will create descriptive summary tables of the extracted data to demonstrate similarities and differences between included studies. We will also provide an overview of the topical interventions and patient wounds.

Where there are multiple studies reporting similar treatment groups and reporting relevant outcomes in a similar fashion we will undertake a meta-analysis using RevMan 5.4.1 if appropriate. It may also be possible to undertake more detailed meta-analyses that allow for different time intervals or subgroups. Pooled mean difference (MD) with 95% confidence interval (CI) will be calculated for continuous data, while relative risk (RR) with 95% CI for dichotomous data will be calculated. We will assess statistical heterogeneity using the I² statistic. If the I² value is more than 50%, a random effects model will be used, whilst for I² values of less than 50%, the fixed effects model will be applied.

The main outcome measure is pain score and we will record this as before-after change in score or as a comparison of scores according to treatment group. Time intervals for recording changes in pain score may differ and we will provide an overview to help identify similar intervals and engage in more detailed analyses. We will record quality of life, cognitive, sleep and other functionality outcomes in addition to adverse events (AEs) in terms of time interval.

In the event that quantitative synthesis is not appropriate for our included studies, we will summarise their findings textually.

***Analysis of subgroups or subsets.*** It may be possible to carry out sub-group analyses comparing different patient or treatment groups, or time intervals depending on the quality of studies included and similarity in outcomes reported.

## Discussion

Currently, wound pain is mostly managed with systemic pharmaceutical agents. However, the ARIW patient panel has expressed a hesitance to take more systemic analgesic drugs for fear of addiction. In addition, persons (often older adults) who live with chronic wounds have at least one co-morbidity such as hypertension, diabetes, or cardiovascular disease, for which they are already taking multiple medications. Background pain in these individuals is common and the use of topical analgesia has the potential to mitigate some of the perceived disadvantages and allay fears associated with systemic agents. There is a lack of understanding in regard to the range of currently available topical agents for managing pain in chronic wounds and their effectiveness. This review will focus on pain management across a range of the most common chronic wounds.

## Dissemination

We will publish the completed review in a peer-reviewed academic journal. We will also make the review available *via* repositories such as The National University of Ireland, Galway’s Access to Research at NUI Galway ( ARAN) facility. We will submit an abstract to an international wound care conference and prepare a summary report prepared for the Journal of Wound Management. We will also report findings of the review to our patient panel and make a video of the results for publication on the website of the ARIW research group. We will also disseminate findings *via* social media sites such as including Twitter and LinkedIn.

## Study status

Review ongoing.

## Additional information

In the event that any changes are made between the protocol and final review, these will be reported in the final review.

## Data availability

### Underlying data

No data are associated with this article.

### Extended data

Open Science Framework: Systematic review of topical interventions for the management of pain in chronic wounds. https://doi.org/10.17605/OSF.IO/WFA64 ^13^.

This project contains the following extended data:

- Wound Pain_Medline search original_18 Dec 21 – word document (search strategy for Medline)

### Reporting guidelines

Open Science Framework: PRISMA-P checklist for ‘Topical interventions for the management of pain in chronic wounds: A protocol for a systematic review’. https://doi.org/10.17605/OSF.IO/WFA64 ^13^.

Data are available under the terms of the Creative Commons Zero "No rights reserved" data waiver (CC0 1.0 Public domain dedication).
