# Supplementary material for: Topical interventions for the management of pain in chronic wounds: A protocol for a systematic review
Source: HRB Open Res. 2022 Aug 19;5:58. [Version 1] doi: 10.12688/hrbopenres.13560.1 (PMC9445559; doi:10.12688/hrbopenres.13560.1)
Supplement: Peer review history

# Peer review: Reviewer response for version 1

**DOI:** 10.21956/hrbopenres.14806.r32718

**Published:** 2022-09-05

**Authors:** Nicoletta Frescos

**Affiliations:** 1 Discipline of Podiatry, School of Allied Health, Human Services and Sport, La Trobe University, Melbourne, VIC, Australia

The protocol for a systematic review on topical interventions for wound related pain will be of interest to the readers involved with treatment of chronic wounds. The topic is worthy of investigation. This is a well written protocol and the authors present a compelling reason as to why this is important research and how it will potentially fill the gaps in knowledge and contribute to evidence-based practice.

 The paper provides a strong rationale and robust methodology. I have a few minor suggestions for consideration that will help with the future systematic review:

1. Pain in diabetes foot ulcers can be challenging to differentiate between wound related pain and neuropathic pain. How will this be addressed?
2. Differentiating between other pain experienced by patients such as chronic wound pain or anticipatory pain. Can these types of pain be treated with topical interventions?

I congratulate the authors on a very well-considered protocol and I look forward to seeing the final SR findings

Is the study design appropriate for the research question?

Yes

Is the rationale for, and objectives of, the study clearly described?

Yes

Are sufficient details of the methods provided to allow replication by others?

Yes

Are the datasets clearly presented in a useable and accessible format?

Yes

Reviewer Expertise:

diabetes foot disease. wound related pain, chronic wound management.

I confirm that I have read this submission and believe that I have an appropriate level of expertise to confirm that it is of an acceptable scientific standard.

# Peer review: Reviewer response for version 1

**DOI:** 10.21956/hrbopenres.14806.r32717

**Published:** 2022-08-23

**Authors:** Terry Treadwell

**Affiliations:** 1 Institute for Advanced Wound Care, Montgomery, AL, USA

It looks very good. I would question the need to use only randomized controlled trials. As long as the paper has good measurements of pain before and after therapy, the information should be usable for the analysis. Since you are including diabetic foot ulcers in the evaluation, has there been any thought given as to how to determine whether the pain is from the wound or from diabetic neuropathy in these patients?  If the pain is due to neuropathy instead of the wound itself, the results in this group may be unreliable. Just a thought. I look forward to seeing your results.

Is the study design appropriate for the research question?

Yes

Is the rationale for, and objectives of, the study clearly described?

Yes

Are sufficient details of the methods provided to allow replication by others?

Yes

Are the datasets clearly presented in a useable and accessible format?

Yes

Reviewer Expertise:

Vascular Surgery,  Management of Acute and Chronic Wounds including all therapies for most all types of wounds.

I confirm that I have read this submission and believe that I have an appropriate level of expertise to confirm that it is of an acceptable scientific standard.
